# Metacognitive evaluation of postdecisional perceptual representations

**DOI:** 10.1167/jov.24.4.2

**Published:** 2024-04-01

**Authors:** Tarryn Balsdon, Valentin Wyart, Pascal Mamassian

**Affiliations:** 1 Laboratoire des Systèmes Perceptifs (CNRS UMR 8248), DEC, ENS, PSL University, Paris, France; 2 Laboratoire de Neurosciences Cognitives et Computationnelles (Inserm U960), DEC, ENS, PSL University, Paris, France

**Keywords:** metacognition, perception, confidence, reverse hierarchy, visual masking, evidence accumulation

## Abstract

Perceptual confidence is thought to arise from metacognitive processes that evaluate the underlying perceptual decision evidence. We investigated whether metacognitive access to perceptual evidence is constrained by the hierarchical organization of visual cortex, where high-level representations tend to be more readily available for explicit scrutiny. We found that the ability of human observers to evaluate their confidence did depend on whether they performed a high-level or low-level task on the same stimuli, but was also affected by manipulations that occurred long after the perceptual decision. Confidence in low-level perceptual decisions degraded with more time between the decision and the response cue, especially when backward masking was present. Confidence in high-level tasks was immune to backward masking and benefitted from additional time. These results can be explained by a model assuming confidence heavily relies on postdecisional internal representations of visual stimuli that degrade over time, where high-level representations are more persistent.

## Introduction

Metacognitive evaluations of perceptual confidence are thought to rely on high-level neural processing (Fleming & Daw, 2017; Vaccaro & Fleming, 2018) that broadcasts confidence as a common currency across sensory modalities (de Gardelle & Mamassian, 2014) and, perhaps, different cognitive tasks (Mazancieux, Fleming, Souchay, & Moulin, 2020). In visual perception tasks, reported confidence partially relies on the quality of the perceptual evidence encoded in visual cortex (Geurts, Cooke, van Bergen, & Jehee, 2022). The information encoded in visual cortex is, therefore, crucial for the computation of confidence. However, it is not yet well-understood how this information in visual cortex is accessed by higher level metacognitive processes. Here, we investigated how metacognitive access to perceptual evidence is affected by the level of visual processing required by the task.

Visual cortex consists of functionally specialized anatomic regions, organized hierarchically (Felleman & Van Essen, 1991) in terms of complexity (Maunsell & Newsome, 1987), receptive field size (Hubel, 1988), and temporal receptive windows (Hasson, Yang, Vallines, Heeger, & Rubin, 2008). These regions are highly interconnected through feedforward, feedback, and lateral projections (Lamme, Supèr, & Spekreijse, 1998). Visual stimulation triggers a fast feedforward sweep of information processing (Nowak & Bullier, 1997), allowing for rapid recognition (Thorpe, Fize, & Marlot, 1996) and identification (Potter, 1976) of the conceptual gist of a visual scene (Kanwisher, 1987). Ongoing neural activity is thought to reflect recurrent processing via feedback and lateral connections with a distinct functional role (Lamme & Roelfsema, 2000): to dynamically change neuronal tuning (Ringach, Hawken, & Shapley, 1997; Cottaris & De Valois, 1998), allowing for contextual modulation (Kapadia, Ito, Gilbert, & Westheimer, 1995; Zipser, Lamme & Schiller, 1996; Lamme & Spekreijse, 1999), and feature integration (Treisman & Gelade, 1980; Treisman, 1986).

Reverse hierarchy theory (Hochstein & Ahissar, 2002) relates the distinction between feedforward and recurrent processing to implicit and explicit visual perception. The initial feedforward sweep provides global scene gist, allowing bottom-up attentional capture by salient features, such as the pop-out of a feature in visual search (Treisman & Gelade, 1980). This feedforward processing is largely implicit, as evidenced by inattentional blindness (Mack & Rock, 1998; Most et al., 2001) and visual masking (Di Lollo, Enns, & Rensink, 2000; Bachmann & Francis, 2013). Explicit vision (or, vision with scrutiny) requires recurrent processing (Pascual-Leone & Walsh, 2001), where attention is focused down to lower level cortical processes in reverse hierarchical order (Ahissar & Hochstein, 2004). Although reverse hierarchy theory is largely supported by studies of perceptual learning and visual search, the theory has general implications for all kinds of perceptual tasks, and the metacognitive self-evaluation of performance in these tasks.

In applying reverse hierarchy theory to metacognition, we predict that high-level perceptual representations should be more readily available for metacognitive scrutiny than low-level representations. How well an observer's confidence reflects the underlying perceptual evidence can be quantified as “confidence efficiency”: the sensitivity of confidence relative to the sensitivity of an “ideal confidence observer,” who relies on exactly the perceptual evidence (Mamassian & de Gardelle, 2022). Confidence efficiency reflects a trade-off between additional noise (such as that incurred with additional noisy processing, impairing confidence sensitivity) (Bang, Shekhar, & Rahnev, 2019; Shekhar & Rahnev, 2021) and additional information not used in the perceptual decision, such as that processed after decision commitment (Pleskac & Busemeyer, 2010; Balsdon, Wyart, & Mamassian, 2020; Balsdon, Mamassian, & Wyart, 2021) or heuristic cues (Maniscalco, Peters, & Lau, 2016). This additional information is referred to as confidence ‘boost’, so that this family of models can be called the confidence noise and confidence boost models (Mamassian & de Gardelle, 2022). In reaching back down the processing hierarchy, the evaluation of low-level perceptual decisions should incur more confidence noise, and perhaps reflect less boost (where additional evidence accumulation could be temporally restricted by longer re-entrant pathways).

We tested this hypothesis by comparing confidence efficiency for high-level and low-level perceptual decisions made on the same stimuli. In Experiments 1 through 4, observers made high-level perceptual decisions about the direction of gaze of an avatar face (Balsdon & Clifford, 2017), or low-level perceptual decisions about the relative contrast of the irises of the eyes. The perception of gaze direction is known to rely on very high-level processing in the visual hierarchy (the superior temporal sulcus) (Calder et al., 2007; Carlin, Calder, Kriegeskorte, Nili, & Rowe, 2011), whereas contrast is processed at very low levels (V1) (Rossi, Rittenhouse, & Paradiso, 1996).

To anticipate our results, we did not find a simple main effect of the level of visual processing, but instead a strong interaction with postdecision time, controlled via the time from stimulus offset to the response cue. This interaction generalized to a fifth experiment using biological motion stimuli, where we compared a high-level task of discriminating walking direction with a low-level decision made on local motion direction. Judging walking direction recruits processing in the superior temporal sulcus (STS) (Puce & Perrett, 2003) and perhaps up to the prefrontal cortex (Koldewyn, Whitney, & Rivera, 2011). In contrast, discriminating local motion direction likely relies on visual motion sensitive areas such as V1 and V5, which have similarly low latencies (Lamme & Roelfsema, 2000). For both types of stimuli, confidence efficiency decreased in the low-level task with more time after perceptual decision commitment, whilst confidence efficiency in the high-level task benefitted from more time after perceptual decision commitment. This interaction implies that the metacognitive evaluation of perceptual decisions is strongly reliant on postdecisional perceptual representations, where high-level representations are more resilient to temporal decay. The interaction can be described computationally by modulating the permeability of the perceptual representation to incoming noise, in an evidence accumulation framework based on a dynamic two-stage signal detection theory (Pleskac & Busemeyer, 2010).

## Methods

We conducted five experiments to understand the effect of visual hierarchical processing level on confidence efficiency, and the interaction with postdecision time. For brevity, Experiment 1 is described in detail, followed by the specific changes made in Experiments 2 through 5. A summary of the methods is presented in Table 1.

**Table 1.** Summary of Experiments.

Experiment	Tested participants	Included participants	Trial pairs	Stimulus	Presentation parameters	Within-subjects comparison	Between-subjects comparison
1	22	20	11,520	Avatar face	White noise mask, response cue 900 ms after stimulus offset	High-level vs. low-level task	N/A
2	116	92	15,364	Avatar face	No mask, response cued 100 ms after stimulus offset	High-level vs. low-level task	N/A
3	136 (low)	93	15,531	Avatar face	Response cued 800 ms after stimulus offset	Mask vs. no-mask	High-level vs. low-level task
	105 (high)	92	15,364				
4	144 (low)	90	15,030	Avatar face	No mask	Response cue at 100 vs. 800 ms	High-level vs. low-level task
	120 (high)	90	15,030				
5	145 (low)	81	13,527	Biological motion	No mask	Response cue at 100 vs. 800 ms	High-level vs. low-level task
	100 (high)	81	13,527				

For each experiment, the table shows the number of participants tested, the number included in the analysis after exclusion, the number of trial pairs for fitting the confidence forced choice model, the stimulus, the critical stimulus presentation parameters maintained throughout the experiment, the within-subjects comparison tested, and any between-subjects comparisons, in the case where two groups of participants were recruited. Experiment 1 was conducted in the laboratory; the rest, online.

All methods were preregistered before data collection, with preregistrations, data, and analysis code (in MATLAB) linked on the main OSF project page^1^ (https://osf.io/6brmt/). Ethical approval was granted by the local ethics committee (CER U-Paris), and the protocols adhere to the Declaration of Helsinki. While the preregistrations were followed in terms of task methodology, sampling plan, and the modelling approach, we note that the preregistered analysis plan allowed for some flexibility with regards to the choice of statistical tests, which we explain in more detail under the analysis section.

### Experiment 1

**Figure 1.**
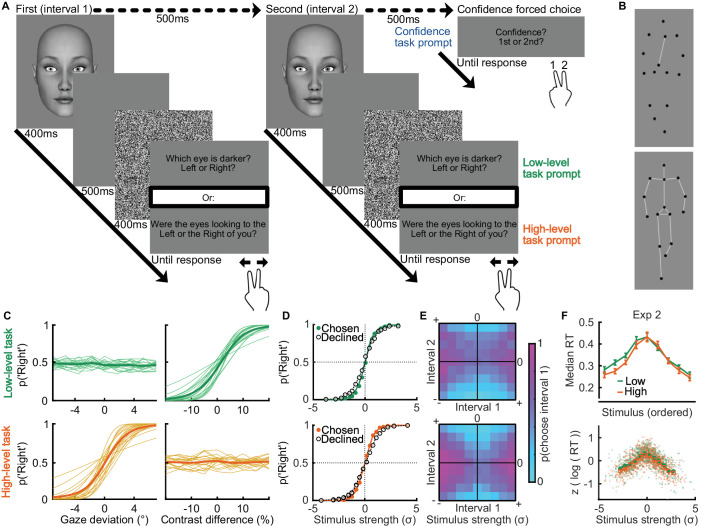
Methods. (**A**) Stimuli for Experiments 1 through 4 and general procedure. On each trial, the observer was presented with a stimulus and asked to make one of two judgments. Judgments were either “which eye is darker? Left or right?” (a low-level task) or “were the eyes looking to the left of right of you?” (a high-level task). In Experiment 1, they were prompted to make their response after a white noise mask. A confidence forced choice judgment was made over two consecutive trials. (**B**) Stimuli for Experiment 5. White lines are added for illustration only and were not part of the stimuli. The stimuli were dots presented with dynamic motion to mimic human walking. In the low-level task (top), the chest and pelvis dots were rotated clockwise or counterclockwise and back to center and the participant was asked to judge the direction, whilst in the high-level task they judged the relative walking direction (to the left or right of directly toward them). (**C**) Individual (thin lines) and average (thick lines) Psychometric functions describing the proportion of rightward responses across presented stimulus strengths. Gaze deviation had no effect on low-level responses and contrast difference had no effect on high-level responses. (**D**) Proportion of rightward responses for trials chosen (filled) as more likely to be correct and trials declined (open) at the forced choice confidence response for the low-level (top) and high-level (bottom) tasks. Stimulus strength was normalized based on the psychometric function of each observer before aggregating across observers. The difference between chosen and declined seems to be small here because most trial pairs contained a similar stimulus difficulty. Confidence efficiency can be better appreciated by comparing choices according to the pairs, as in (**E**). (**E**) Proportion of trial pairs in which the first decision was chosen as more likely to be correct, by normalized stimulus strength across the two intervals for the low-level (top) and high-level (bottom) tasks. The more clearly cyan (0) and magenta (1) are divided along the diagonal, the more confidence is likely to discriminate correct from incorrect perceptual decisions. (**F**) Reaction times by stimulus strength in each task from Experiment 2. The top shows the average and 95% within subjects confidence (error bars) median reaction times by stimulus strength ordered as in (**C**). The consistency across tasks and observers can be better appreciated by the bottom, where stimulus strength is normalized by each individuals psychometric function, and reaction times are normalized by taking the difference from the mean (across both tasks) in units of standard deviation (*z*-scored) of the logarithm of reaction times. Individuals are shown in small markers, and the average in large markers.

Participants were recruited to the laboratory for two experimental sessions of 1 hour each. In both sessions, they completed a block of the high-level task and a block of the low-level task (in counterbalanced order). For both tasks, stimulus presentation was the same: a fixation cross was presented for 400 ms, followed by an avatar face stimulus for 400 ms, followed by a 400 ms white noise mask after a 500 ms blank, and then the response was cued with the words “left or right?” (Figure 1A). Stimuli were presented on a gamma corrected ViewSonic 21” monitor, 57 cm from the participant, running at 60 Hz with resolution of 1,280 × 720 pixels, using MATLAB and the psychophysics toolbox (Brainard, 1997; Pelli, 1997; Kleiner, Brainard, & Pelli, 2007). The avatar face was female, with cropped hair and a neutral expression (created with Daz software; daz3d.com). The image was grayscale, presented at 75% contrast against a grey background (mean luminance 60 cd/m^2^), subtending 14° of visual angle. The original eyes were replaced with realistic counterparts to manipulate the direction of gaze and relative contrast of the irises according to precise angular coordinates and gray levels. The high-level task was to discriminate left from right gaze direction (with random contrast difference), and the low-level task was to discriminate whether the left or right eye was “darker” (higher contrast, with random gaze directions), using the left and right arrow keys of a standard keyboard.

The method of constant stimuli was used, with gaze deviations [0, 1.75, 3.5, 5.25, 7] (degrees rotated horizontally to the left and right of direct gaze), and contrast levels [0, 5, 10, 15, 20] (percent difference in Michelson contrast between left and right eye). In Experiment 1 (but not Experiments 2–5) the task-relevant and task-irrelevant cues were manipulated in both tasks, such that observers were presented with the same set of stimuli in the high-level and low-level tasks. The task-irrelevant cue had no effect on performance: Figure 1C shows the proportion of rightward responses in the low-level task remained around 0.5 across gaze deviations, and similarly, the proportion of rightward responses in the high-level task remained around 0.5 across differences in relative iris contrast. Fitted psychometric functions produced flat slopes of −0.016 and −0.001 on average for the task irrelevant cue but slopes of 0.42 and 0.41 (as a function of stimulus range) for the task-relevant cue in the low- and high-level tasks, respectively.

The stimulus levels were chosen according to a pilot study to generate approximately equal performance across tasks. As presented more fully in the Results, we found evidence in favor of the null hypothesis of no difference in perceptual sensitivity across the low- and high-level tasks. In addition, in Experiment 2 we were able to measure perceptual decision reaction times, and found no difference in reaction times across tasks, suggesting the tasks were similarly effortful (Klein & Yadav, 1989). Figure 1F shows median reaction times decreased with stronger stimulus evidence for the perceptual decision, and this function completely overlaps across the two tasks. This suggests the stimulus levels were adequately chosen to equate the two tasks in terms of the perceptual decision.

Confidence efficiency was measured using the confidence forced-choice task (Mamassian, 2020): every two perceptual decisions the observer chose which decision they thought was more likely to be correct (using the 1 and 2 keys to indicate the first or second decision). Across stimulus strengths, trials chosen as more likely to be correct should follow a steeper psychometric function than the trials not chosen (declined) (de Gardelle & Mamassian, 2015). This is demonstrated in Figure 1D, where responses have been aggregated across participants after normalizing stimulus strength by individual sensitivity (more details below), with declined trials on average showing proportions of rightward responses closer to 0.5. However, confidence choices depend on both stimulus strengths presented in the pair of trials (Mamassian & de Gardelle, 2022): observers should choose the trial with more evidence in favor of their perceptual decision, which is on average the trial with greater stimulus strength. This is demonstrated in Figure 1E, which shows observers are more likely to choose interval 1 (the first trial in the pair) when the stimulus strength of interval 1 is greater than interval 2.

The stimulus strengths presented in each pair were manipulated to improve the cost–benefit analysis of confidence efficiency: because pairs of stimuli that differ greatly in difficulty lead to ceiling confidence judgments, their number was decreased. Consequently, four pairs of trials of each combination of stimulus strength were presented, plus an additional eight pairs of trials of matched stimulus strength (unsigned), plus an additional four pairs of trials at stimulus strengths that differ by just one level, making altogether 1,152 trials (576 pairs for the confidence judgment). The number of participants was determined based on the number of pairs of trials required to fit the parameters of the confidence forced-choice model at the group level (≤10,000) (Mamassian & de Gardelle, 2022). We initially recruited 20 participants, and replaced one participant who did not return for the second session, and one whose perceptual responses did not vary with the presented stimulus (further details on exclusion criteria in the Analysis).

### Experiments 2 through 5

Experiments 2 through 5 were conducted with participants recruited online (using the Prolific Academic online platform, prolific.co). Participants were instructed to complete the experiment on a desktop or laptop device (tablets and smartphones forbidden), at a comfortable distance, in a quiet environment. Stimulus presentation was controlled using PsychoPy (Peirce et al., 2019), hosted on Pavlovia (pavlovia.org). Stimulus size was controlled using the credit card method (Li, Joo, Yeatman, & Reinecke, 2020) and screen luminance was estimated by asking participants to report the minimum visible white and black contrast. The number of trials was reduced to 334 per task (167 pairs), so that the experiment could be completed within 40 minutes. To decrease the number of images, the task-irrelevant stimulus feature was kept neutral, that is, observers were presented with stimuli with direct gaze in the low-level task and equal iris contrast in the high-level task in Experiments 2 through 4. We expected online participants to provide more noisy data than participants recruited to the laboratory. We therefore aimed to include at least 80 participants (13,360 pairs of trials), by first recruiting 100 participants and then replacing those who met the exclusion criteria until at least 80 could be included.

In Experiment 2, stimulus presentation was the same, but the mask was removed. Instead, the response cue was presented 100 ms after stimulus offset. Our initial motivation for this change was to decrease the duration of the experiment and the complexity of stimulus presentation for online observers, who may be annoyed by the backward masking procedure. This experiment was meant to simply generalize the findings of Experiment 1 to a larger population.

The results of Experiment 2 led us to run Experiments 3 and 4. In Experiment 3, two groups of participants were recruited. One group completed the low-level task, and the other the high-level task. Within participants we compared the effect of the white noise mask: in separate blocks, the stimuli were either presented in a similar manner as Experiment 1 (mask condition, with 400 ms of blank followed by 400 ms of white noise mask), or the response was cued after 800 ms of blank (no-mask condition). The group performing the low-level task were recruited first, and this was preregistered as a separate experiment: had we not found the mask to significantly decrease confidence efficiency in the low-level task, we would not have tested the effect in the high-level task.

Experiment 4 compared the effect of response cue timing: in separate blocks, the response cue was presented 100 ms after stimulus offset (as in Experiment 2) or 800 ms after stimulus offset (as in the no-mask condition of Experiment 3). One group of participants performed the low-level task and a second group performed the high-level task.

Experiment 5 was the same as Experiment 4, but used different stimuli. Instead of avatar faces, observers were presented with 400 ms of biological motion stimuli, formed of 15 black dots displaced to mimic a human walking (based on the neutral gender walker of Troje, 2002) (Figure 1B). In the high-level task, observers discriminated whether the walking direction was leftward or rightward of directly toward them (presented at [0, 4, 8, 14, 20] degrees left and right of direct). A separate group of participants completed the low-level task, discriminating whether the chest and pelvis dots (both located near the vertical midline) rotated leftward (counter-clockwise) or rightward (clockwise) and back to center (with [0, 1, 2, 3, 4] pixels of sinusoidal motion relative to the original image size of ∼980 pixels in height). These stimulus levels were chosen according to a pilot study to generate approximately equal performance across tasks. The starting position of the stimuli was pseudorandomly chosen on each trial.

### Analysis

The proportion of rightward responses across the presented stimulus levels were fit with a psychometric (cumulative Gaussian) function for each observer. The slope of the psychometric function (standard deviation of the cumulative Gaussian) was taken as an index of perceptual sensitivity. We initially preregistered that “Participants will be rejected with replacement if their performance fails to increase above chance in either of the T1 perceptual tasks”; we note this was not specific. Observers were excluded if the slope exceeded one-half of the range of stimulus values (meaning their responses did not vary with the stimulus). After looking at the confidence responses in the online data from Experiment 2, we additionally chose to exclude participants who displayed large biases in their confidence choices (used one confidence response on more than 78% of trials, representing a significant difference from unbiased based on Experiment 2, resulting in a total of 14 participants excluded across all 888 participants from the five experiments). The stimulus values were then recentered around the perceptual response criterion (mean of the cumulative Gaussian) and rescaled by the slope (units of standard deviation), such that the values for each participant were normalized to their perceptual response function. The confidence forced-choice model (Mamassian & de Gardelle, 2022; github.com/mamassian/cfc) was used to quantify confidence noise, boost, and confidence efficiency in data aggregated across subjects. This model quantifies confidence efficiency as a measure of how well the observer makes confidence decisions relative to their perceptual decisions, by assessing performance in confidence choices relative to the “ideal confidence observer,” who uses exactly the evidence of the perceptual decision to make their confidence decision. The human observer can differ from the “ideal confidence observer” in two ways: additional confidence noise, such as that incurred during additional processing (impairing confidence efficiency), and additional boost, which captures an improvement in confidence efficiency via the use of additional information not used in the perceptual decision (such as evidence accumulated after perceptual decision commitment).

This modelling analysis was conducted as described by Mamassian and de Gardelle (2022), using the toolbox provided github.com/mamassian/cfc. Briefly, the model assumes that, in line with classical psychophysics, the physical stimulus provides some evidence strength, µ_*s*_, and the observer has a noisy sensory representation of this evidence, *s*, on which to base their perceptual decisions

(1) $$\begin{array}{c} s=\mu_{s}\;+\;\varepsilon_{s}\; \end{array}$$

where ϵ_*s*_ is drawn from a zero-mean Gaussian with variance $\sigma_{s}^{2}$. The ideal confidence observer is defined as relying on this same evidence, normalised to the perceptual response criterion, *c*, and sensory noise, σ_*s*_, to form the confidence evidence, *w*, upon which a confidence judgment is made

(2) $$\begin{array}{c} w_{ideal}=\frac{s-c}{\sigma_{s}}.\; \end{array}$$

The model proposes that the confidence evidence of human observers is affected by additional confidence noise, ϵ_*c*_, which is drawn from a zero-mean Gaussian with independent variance, $\sigma_{c}^{2}$

(3) $$\begin{array}{c} w_{human}=\frac{s-c}{\sigma_{s}}+\;\varepsilon_{c}\;.\; \end{array}$$

Human observers can also integrate additional evidence not used for the perceptual decision, which is modelled with the confidence boost parameter, α, representing the proportion of additional evidence

(4) $$\begin{array}{c} w_{human}=\frac{\alpha \mu_{s}+\left(1-\alpha \right)s-c}{\sigma_{s}}+\varepsilon_{c}.\; \end{array}$$

The balance of confidence noise and confidence boost determines how well the observer's confidence reflects their perceptual accuracy. This match between confidence and accuracy is measured as confidence efficiency, the ratio of the equivalent noise affecting the observers’ confidence compared with the ideal confidence observer. The variance of the equivalent noise, τ^2^ (a fitted parameter), attributes all the noise in the confidence evidence as confidence noise (forcing the confidence boost to be 1), so that confidence efficiency is defined as

(5) $$\begin{array}{c} \eta =\frac{\tau_{ideal}^{2}}{\tau_{human}^{2}}.\; \end{array}$$

In this way, confidence efficiency gives a measure of confidence sensitivity that is independent of perceptual sensitivity (Mamassian & de Gardelle, 2022). The model was fit by aggregating the data across participants (after normalizing their perceptual response functions). The sampling distribution of the confidence parameters (efficiency η, confidence noise σ_*c*_, and confidence boost α) was estimated by bootstrapping (1,000 permutations of resampled participants).

Our preregistered analysis plan was flexible with respect to the choice of statistical test. We were concerned standard *t* tests may be inappropriate, because the parameter values have a lower bound at 0. We preregistered that we would use Wilcoxon sign-rank tests, unless the data were sufficiently normally distributed, and Bayesian statistics would be used to examine evidence for the null. A Kolmogorov–Smirnov test suggested the data could be approximated by a normal distribution (in Experiment 1, confidence efficiency Kolmogorov–Smirnov statistic = 0.013, *p* > 0.99; confidence noise Kolmogorov–Smirnov statistic = 0.022, *p* = 0.72; confidence boost Kolmogorov–Smirnov statistic = 0.039, *p* = 0.1; and Experiment 2, confidence efficiency Kolmogorov–Smirnov statistic = 0.014, *p* > 0.99; confidence noise Kolmogorov–Smirnov statistic = 0.02, *p* = 0.80; confidence boost Kolmogorov–Smirnov statistic = 0.02, *p* = 0.78). So, we initially used *t* tests; however, it became more obvious that it would be beneficial to make inferences about the probability of the null hypothesis of no difference between conditions, so we switched to Bayesian statistics across all comparisons for consistency. We note that the same conclusions were drawn from the *t* tests and the Bayesian statistics (with the exception of accepting the null in some Bayesian comparisons, which produced large *p* values for the *t* tests).

The sample distribution was analysed using hierarchical Bayesian models (Wagenmakers, Lodewyckx, Kuriyal, & Grasman, 2010), by taking *N* quantiles of the bootstrapped data, where *N* is the number of participants. The models assumed a gamma distribution for confidence efficiency and confidence noise, and a beta distribution for confidence boost. The within-subjects comparison assumed that each subject had some effect, *x*, such that their value in condition *B* differs from condition *A* by *x*. Across subjects, the effects, *a,* are normally distributed with mean µ_*x*_ and variance $\sigma_{x}^{2}$, such that the effect size, δ, is $\delta =\;\frac{\mu_{x}}{\sigma_{x}}$. Between-subject comparisons modelled δ directly, where the difference in the group means, *y*, is *y* =  δ × σ_*AB*_, with $\sigma_{AB}^{2}$, computed as the combined variance across the two groups. The posterior was estimated by Markov chain Monte Carlo simulation (with 12,000 samples over three independent chains, 1,000 samples burn-in and three samples thinning), using the slice sampling method (Neal, 2003) implemented in MATLAB. For all parameters, uninformative priors were specified as a uniform distribution over all possible (or a large range) of parameter values. Evidence in favor of the alternative hypothesis (δ ≠ 0) was based on the 95% highest density interval of the posterior distribution of δ values. Bayes factors were computed based on the Savage–Dickey ratio (Wagenmakers et al., 2010) using a unit information prior (Kass & Wasserman, 1995).

**Figure 2.**
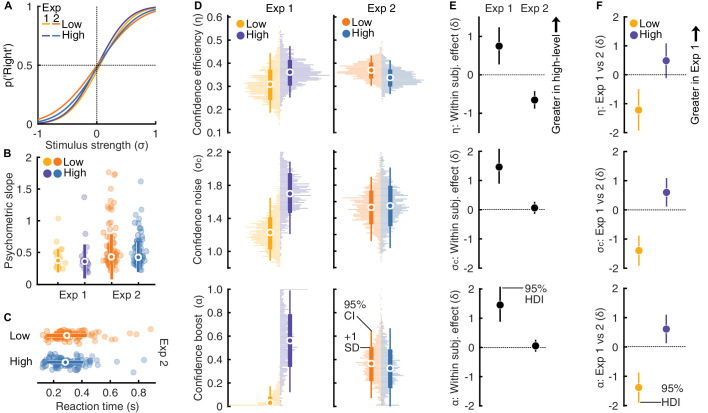
Results of Experiments 1 and 2. (**A**) Psychometric functions averaged over participants in Experiments 1 and 2. (**B**) Comparison of slopes of individual participants, solid markers show the group median, and vertical lines show ±1 standard deviation. (**C**) Median reaction times in Experiment 2 for individual participants, where solid markers show the group median, and horizonal lines show ±1 standard deviation. (**D**) Confidence efficiency (top row), confidence noise (middle), and confidence boost (bottom) in Experiment 1 (left column) and 2 (right). Markers show the mean, thin vertical lines show 95% Confidence Intervals, thick show ±1 SD, and horizontal histograms represent the sample density from bootstrap resampling. (**E**) Within subjects effect size for the difference in confidence efficiency (top), noise (middle), and boost (bottom), comparing the high-level and low-level tasks in Experiment 1 (left symbol) and 2 (right) based on hierarchical Bayesian modelling. Markers show the posterior mean and vertical line shows the 95% highest density interval. (**F**) Between subjects effect size for the difference in confidence efficiency (top), noise (middle), and boost (bottom), comparing the low- (left symbol) and high-level (right) tasks in Experiments 1 and 2. Markers show the posterior mean and vertical lines show the 95% highest density interval.

The same process was used to analyze participants’ perceptual sensitivity (the slope of the psychometric function over the range of stimulus values), and reaction times, but the raw samples were used instead of inferring the samples from the bootstrapped distribution. Only reaction times in conditions where there was a short duration (100 ms) between stimulus offset and the response cue were used in the analysis of reaction times, as the response cue was presented soon enough that the reaction time provided an estimate of decision time that was unlikely to have been corrupted by additional time waiting for the cue. Figure 1F shows how reaction times depend on the stimulus strength, suggesting that they do reflect the difficulty of the decision, and Figure 2C shows the reaction times for individual participants were always more than 100 ms after the cue. Median reaction times were calculated for each participant using the half-block (167 trials) of responses closest to the middle of the experiment: reaction times tended to decrease over the course of the experiment, so participants who performed the short condition in the first block would appear to have longer reaction times than participants who performed the short condition second. The data from Experiment 2 suggested taking the half-block closest to the middle of the experiment effectively dealt with this possible confound.

## Results

### Perceptual decisions in the high-level and low-level tasks

Although our measure of confidence efficiency accounts for potential differences in underlying perceptual sensitivity, we endeavored to minimize any differences in perceptual decision-making behavior between the high-level and low-level tasks. The values of the high-level and low-level stimulus cues were chosen based on pilot data to give approximately equal performance. We confirmed that perceptual sensitivity was approximately equal in the two tasks by comparing the slope of the psychometric function over the range of stimulus values (Figure 1C). In Experiment 1, the estimated posterior effect size (δ) of the difference in sensitivity was small *E*(δ | *x*_*high* − *low*_) = 0.03 and the 95% highest density interval overlapped with 0 [−0.40; 0.46], suggesting little evidence for the alternative hypothesis of a difference in sensitivity. We computed a Bayes factor of evidence in favor of the alternative hypothesis using the Savage–Dickey ratio (Wagenmakers et al., 2010) with a unit information prior (Kass & Wasserman, 1995). We found BF_10_ = 0.21; computed in this direction, the larger the number relative to 1, the more evidence against the null hypothesis (and the smaller the number relative to 1 the more evidence in favor of the null). In general, a Bayes factor of greater than 3.2 (<0.31) could be considered substantial evidence in favor of the alternative hypothesis (null hypothesis) (Kass & Raftery, 1995). We therefore show evidence in favor of the null hypothesis of no difference in sensitivity across the high-level and low-level tasks.

We additionally confirmed there was no interaction between the task-relevant stimulus cues by showing flat psychometric functions across cross-task cues (slope on the probability of a rightward response in the low-level task based on normalized gaze direction = −0.016; and for gaze direction judgments based on relative iris contrast, slope = −0.001 (Figure 1C). That is, relative iris contrast had no influence on gaze direction responses in the high-level task, and gaze direction had no influence on iris contrast responses in the low-level task. In Experiments 2 through 5, the task-irrelevant cue was kept neutral.

In Experiment 2, we also found little evidence of a difference in sensitivity across the two tasks (*E*(δ | *x*_*high* − *low*_) = −0.18 [−0.38; 0.03] 95% highest density interval; BF_10_ = 0.43). The average psychometric functions from Experiments 1 and 2 are presented in Figure 2A, with the slopes of individual participants shown in Figure 2B. In addition, we performed an exploratory analysis examining median reaction times across the high-level and low-level tasks and found no evidence for a difference (*E*(δ | *x*_*high* − *low*_) = −0.13 [−0.08; 0.34]; BF_10_ = 0.23; Figure 2C).

Together, this evidence suggests perceptual decision-making behavior was very similar across the two tasks. Any differences in the confidence efficiency cannot be explained by differences in the underlying perceptual decisions.

### Confidence efficiency for high-level and low-level perceptual decisions

Our main hypothesis was that confidence efficiency would be greater for high-level compared with low-level perceptual decisions, based on reverse hierarchy theory. Confidence efficiency is the sensitivity of confidence decisions relative to that expected if observers had used exactly the same evidence for confidence as their perceptual decisions. This measure of metacognitive sensitivity accounts for the underlying perceptual sensitivity. In addition, we examined confidence noise (additional noise impairing confidence sensitivity) and confidence boost (the use of additional information that improves confidence sensitivity), which trade-off in contributing to confidence efficiency. Experiment 1 offered initial support for the hypothesis that confidence efficiency is greater for high-level perceptual decisions than low-level perceptual decisions: we found greater confidence efficiency in the high-level task compared with the low-level task (*E*(δ | *x*_*high* − *low*_) = 0.75 [0.27; 1.24] 95% highest density interval; BF_10_ = 32.26) (Figure 2D and E, top left). Comparisons of confidence noise and confidence boost suggested that, although low-level confidence efficiency was impaired by relatively less noise (*E*(δ | *x*_*high* − *low*_) = 1.46 [0.89; 2.08]; BF_10_ >1,000) (Figure 2D and 2E, middle left), low-level confidence also showed little sign of confidence boost, which substantially contributed to high-level confidence efficiency (*E*(δ | *x*_*high* − *low*_) = 1.95 [1.24; 2.7]; BF_10_ 1,000) (Figure 2D and E, bottom left). In this experiment, participants were presented with the same sets of stimuli across the two tasks, they showed similar sensitivity with their perceptual decisions across the two tasks, and yet confidence efficiency was significantly superior for the high-level perceptual decisions.

In Experiment 2, we attempted to replicate the results of Experiment 1 in a larger sample of participants recruited online. We had preregistered one-tailed tests; however, the effect on confidence efficiency in Experiment 2 was the opposite of that predicted: confidence efficiency was greater in the low-level task compared with the high-level task (*E*(δ | *x*_*high* − *low*_) = −0.66 [−0.42; −0.88]; BF_01_ >1,000) (Figure 2D and E, top right).

We performed post hoc (not preregistered) comparisons to examine the differences between Experiments 1 and 2. Perceptual decision performance was overall similar in Experiment 2 compared with Experiment 1: the slopes of the psychometric functions (reflecting perceptual sensitivity) were not substantially dissimilar in the high-level task (*E*(δ | *x*_*Exp* 1 − 2_) = 0.31 [−0.25; 0.8]; BF_10_ = 0.55) (Figure 2D), although slightly lower in the low-level task (*E*(δ | *x*_*Exp* 1 − 2_) = 0.48 [0.09; 0.8]; BF_10_ = 4.35). Confidence efficiency was greater in the low-level task of Experiment 2 (*E*(δ | *x*_*Exp* 1 − 2_) = −1.22 [−0.5; −1.92]; BF_10_ = 25; but not substantially dissimilar in the high-level task; *E*(δ | *x*_*Exp* 1 − 2_) = 0.49 [−0.12; 1.08]; BF_10_ = 0.93) (Figure 2F, top). Performance metrics were not overall worse in Experiment 2, this finding suggests that the difference in the results is not due to differences in stimulus presentation or task engagement in the online format.

### The effect of visual masking on confidence efficiency

**Figure 3.**
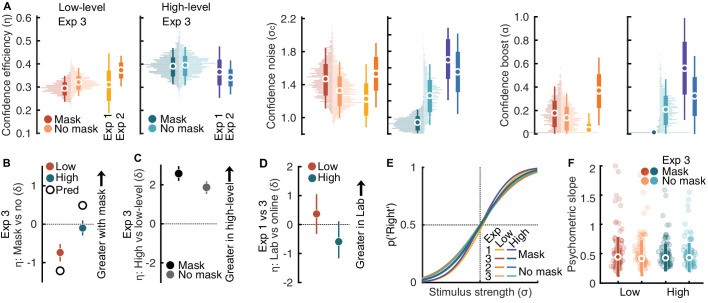
Effects of late visual masking. (**A**) Confidence efficiency (left columns), confidence noise (middle), and confidence boost (right) in the low- and high-level tasks (left and right plots). Horizontal histograms show the sample distribution estimated by bootstrapping, the means are shown in the markers, and 95% CIs with thin vertical lines and ±1 SD with thick. The results of Experiments 1 and 2 are shown adjacent for comparison. (**B**) Within-subject effect size of the mask on confidence efficiency. The colored markers show the posterior mean and vertical lines show the 95% highest density interval. The open black markers show the between subjects comparisons from Experiments 1 and 2 for the same conditions. (**C**) Between subjects effect comparing the high-level and low-level tasks in the mask and no-mask conditions of Experiment 3, vertical lines show the 95% highest density interval. (**D**) Between-subjects effect sizes comparing confidence efficiency in Experiment 1 (laboratory experiment) and the mask condition of Experiment 3 (online experiment) for the low- and high-level tasks (different participants in Experiment 3) vertical lines show the 95% highest density interval. (**E**) Average psychometric functions across all Experiments and conditions. (**F**) Slope of the psychometric functions for each participant in Experiment 3, the large markers show the group medians, and vertical lines show ±1 standard deviation. Colors correspond with (**A**).

We questioned whether there was some difference between the tasks of Experiments 1 and 2 that could explain the reversal of the effect on confidence efficiency. In Experiment 1, a white noise mask was presented after the stimulus, but this was removed in Experiment 2, with the response cue presented 100 ms after the stimulus. The white noise mask would activate processing in the same cortical regions as the low-level representation of relative iris contrast and could potentially affect very late processing for confidence whilst having limited effect on the earlier perceptual decision processes. Experiment 3 compared the effect of the mask within-subjects, keeping the duration between stimulus offset and response cue stable (800 ms). As predicted, there was an effect of the mask on confidence efficiency in the low-level task (*E*(δ | *x*_*mask* − *nomask*_) = −0.73 [−0.51; −0.97]; BF_10_ >1,000) (Figure 3A, left), whereas perceptual sensitivity was unaffected (*E*(δ | *x*_*mask* − *nomask*_) = 0.05 [−0.25; 0.16]; BF_10_ = 0.12) (Figures 3E and F). This was mainly due to a decrease in confidence noise in the no-mask condition (*E*(δ | *x*_*mask* − *nomask*_) = 0.54 [0.32; 0.76]; BF_10_ >1,000) (Figure 3A, middle) with some evidence for a decrease in confidence boost (*E*(δ | *x*_*mask* − *nomask*_) = 0.27 [0.06; 0.47]; BF_10_ = 3.13; although both conditions showed close to no boost) (Figure 3A, right).

In the high-level task, the mask had no effect on perceptual sensitivity (*E*(δ | *x*_*mask* − *nomask*_) = 0.02 [−0.19; 0.22]; BF_10_ = 0.11) nor confidence efficiency (as predicted; *E*(δ | *x*_*mask* − *nomask*_) = 0.1 [−0.29; 0.1]; BF_10_ = 0.16) (Figure 3A). The confidence noise and boost parameters did appear to differ between mask and no-mask conditions in the high-level task (despite the effects cancelling to give equal confidence efficiency; there was more confidence noise in the no-mask condition; *E*(δ | *x*_*mask* − *nomask*_) = −1.71 [−2.02; −1.38]; BF_10_ > 1,000; and more confidence boost; *E*(δ | *x*_*mask* − *nomask*_) = −1.56 [−1.88; −1.25]; BF_10_ >1,000) (Figure 3A).

**Figure 4.**
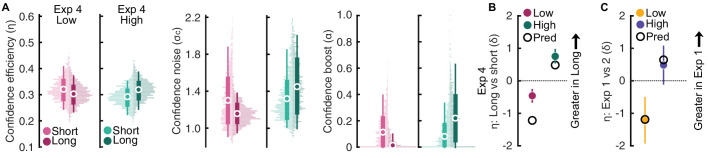
Interaction between decision time and response cue time. (**A**) Confidence efficiency (left), confidence noise (middle), and confidence boost (right) in Experiment 4, in the low-level (magenta) and high-level (green) tasks. Thin vertical lines show 95% CI, thick show ±1 SD. (**B**) Within-subjects effects (short vs long duration to response cue) in the low- and high-level tasks in Experiments 4, vertical lines show the 95% highest density interval, open black markers show the predicted effect size comparing Experiments 1 and 2. (**C**) Effect size of the difference in confidence efficiency between Experiments 1 and 2 (colored markers), against the prediction based on the additive effects of the mask and duration to response cue from Experiments 3 and 4 (open black markers).

We performed some post hoc (not preregistered) analyses to assess whether the mask explained the differences between Experiments 1 and 2. Critically, the effect of the mask was not sufficient to explain the difference between Experiments 1 and 2. In the low-level task, the between subjects effect on confidence efficiency was *E*(δ | *x*_*Exp* 1 − 2_) = −1.22 [−1.91; −0.41]; BF_10_ = 25), compared with the within-subject effect *E*(δ | *x*_*mask* − *nomask*_) = −0.73 in Experiment 3 (Figure 3B); we would expect the effect size to be at least as large (e.g., Figure 4C).

Interestingly, and in agreement with the results of Experiment 1, confidence efficiency was greater for the high-level task in both the mask and no-mask conditions of Experiment 3 (Figure 3C). Although the mask condition closely mirrored Experiment 1, the no-mask condition differed substantially from Experiment 2 (Figure 3D). Had the mask been completely responsible for the decrease in low-level confidence efficiency in Experiment 1 compared with Experiment 2, confidence efficiency should have recovered in the no-mask condition of Experiment 3, and produced similar confidence efficiency as Experiment 2. Therefore, the presence or absence of a mask is not sufficient to explain the different results in Experiments 1 and 2.

### The effect of response cue timing on confidence efficiency

Although the mask explained some of the difference in effects on confidence efficiency between Experiments 1 and 2, it was clearly not the only explanation. The other difference between Experiment 1 and Experiment 2 was the duration between stimulus offset and the response cue, which could be termed “poststimulus time.” In Experiments 1 and 3, the response cue was presented 900 ms and 800 ms, respectively, after stimulus offset, where observers likely committed to their perceptual decisions long before the cue. In Experiment 2, the response cue was presented just 100 ms after stimulus offset, with the next stimulus or confidence decision cued 200 ms after the response. We tested whether the timing of the response cue could explain some of the difference between Experiment 1 and Experiment 2 in Experiment 4, by comparing conditions with a short (100 ms) and long (800 ms) duration between stimulus offset and response cue (within participants, and with separate groups of participants performing the low- and high-level tasks).

In the low-level task, there was a significant decrease in confidence efficiency with longer duration between stimulus offset and the response cue (*E*(δ | *x*_*short* − *long*_) = 0.46 [0.25; 0.68]; BF_10_ >10,001) (Figure 4A, left), and underlying this was a decrease in noise (*E*(δ | *x*_*short* − *long*_) = 0.56 [0.34; 0.77]; BF_10_ >1,000) (Figure 4A, middle), and a greater decrease in boost (*E*(δ | *x*_*short* − *long*_) = 0.82 [0.58; 1.06]; BF_11_ >1,000) (Figure 4A, right). In the high-level task, there was a significant increase in confidence efficiency with longer duration between stimulus offset and the response cue (*E*(δ | *x*_*short* − *long*_) = −0.75 [−0.98; −0.53]; BF_11_ > 1,000) (Figure 4A, left), with increased noise (*E*(δ | *x*_*short* − *long*_) = −0.48 [−0.7; −0.26]; BF_10_ >1,000) (Figure 4A, middle), and a larger increase in boost (*E*(δ | *x*_*short* − *long*_) = −0.75 [−0.99; −0.52]; BF_10_ > 1,000) (Figure 4A, right). This finding was in line with our prediction that response cue timing interacts with the effect of task on confidence efficiency, increasing confidence efficiency with more time in the high-level task and decreasing in the low-level task.

Examining the effect sizes (not preregistered), the difference in confidence efficiency between the short and long duration conditions was not sufficient to explain the difference between Experiments 1 and 2 in the low-level task, but was sufficient in the high-level task (Figure 4B). However, adding the effects from Experiment 3 and 4 (prediction in open markers in Figure 4C) almost perfectly captures the effect size of the difference in confidence efficiency between Experiments 1 and 2 (Figure 4C). That is, the different effects on confidence efficiency between Experiments 1 and 2 can be explained by the additive effects of the mask and the duration from stimulus offset to response cue.

### Postdecision processes and confidence efficiency

**Figure 5.**
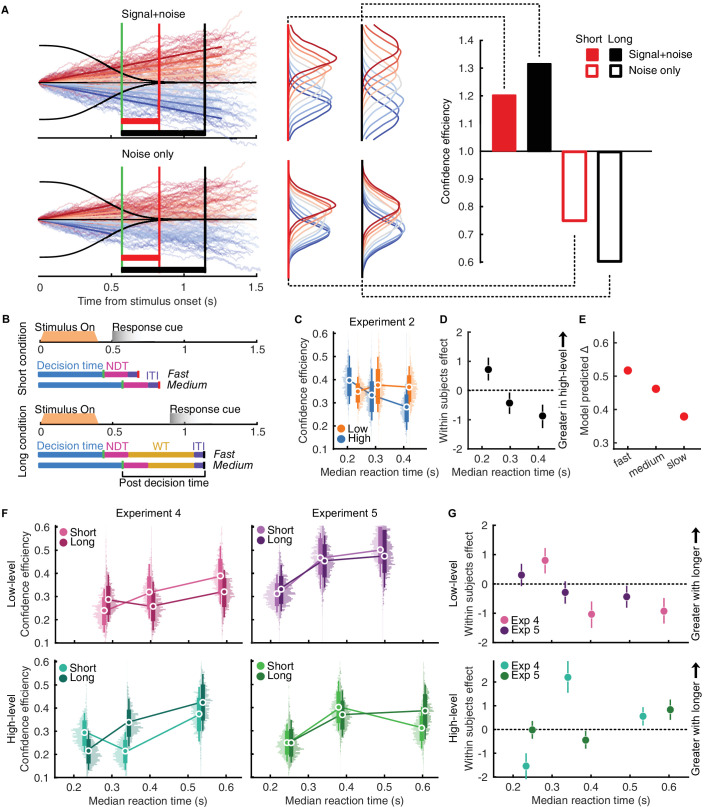
Perceptual evidence accumulation for decisions and confidence. (**A**) Simulations of perceptual evidence accumulation and the effect of long and short response cues. The sequential sampling process (Equation 6) is shown to the left, where evidence is accumulated up to a collapsing bound (thick black curves). The median decision time is shown with the green line in these plots. This demonstration is based on the parameters fit to the median reaction time observers from Experiment 4. The red and black boxes correspond with the median postdecision time (≤200 ms after the response, when the next decision is cued) in the long and short duration conditions respectively. The top plot demonstrates continued accumulation with the same signal-to-noise ratio as predecision commitment, the bottom plot shows continued accumulation of noise only (no additional signal evidence after decision commitment). The distributions of final accumulated evidence (for each stimulus strength) are shown to the right, assuming accumulation continues until the next trial in the short and long duration to response cue conditions. These distributions of final accumulated evidence for confidence generate different predictions about confidence efficiency, shown in the bar plot to the right. Continued accumulation of the same signal + noise as prior to decision commitment (filled bars) predicts an increase in confidence efficiency with more time, whereas accumulating noise only predicts a decrease in confidence efficiency (open bars). (**B**) Demonstration of the relationship between postdecision time and decision time in the short (top) and long (bottom) duration to response cue conditions. The blue bars represent decision time, pink, nondecision time (NDT), and purple, the intertrial interval (ITI). In the long duration condition there is additional waiting time (WT, yellow) where the participant waits for the response cue. Assuming perceptual decision processes proceed in the same manner in both conditions (as there was no substantial difference in perceptual sensitivity), participants who made decisions faster (labelled ‘fast’) would have relatively longer postdecision time (NDT + WT) in the long duration condition compared with those who took longer (labelled “medium”). (**C**) Confidence efficiency in Experiment 2, splitting participants into three equal groups based on their reaction times. Thin error bars show 95% CI, thick show ±1 SD. (**D**) Within subjects effects for the difference in high-level and low-level confidence efficiency in (**C**). Error bars show 95% highest density interval. (E) Model predicted difference in confidence efficiency between the high-level and low-level tasks assuming confidence efficiency is based on continued accumulation with the same signal-to-noise ratio as the prior to decision commitment in the high-level task, and continued accumulation of noise only in the low-level task. The pattern of decreasing difference is similar to that of the data in Experiment 2. (**F**) Confidence efficiency in the low-level (top) and high-level (bottom) tasks for participants split into three RT groups based on their reaction times in the short duration condition in Experiments 4 (left) and 5 (right). Thin error bars show 95% CI, thick show ±1 SD. (**G**) Within-subjects effects for the difference between the short and long duration to response cue conditions in Experiments 4 and 5. Error bars show 95% highest density interval.

The presentation of the mask, and the manipulation of response cue timing, occur long after participants have likely committed to their perceptual decisions (and indeed these manipulations had little effect on perceptual sensitivity). We therefore hypothesized that these effects on confidence efficiency might be driven by postdecision processes. Two-stage dynamic signal detection theory (Pleskac & Busemeyer, 2010) proposes that confidence is based on ongoing evidence accumulation, which continues after perceptual decision commitment, thus providing one possible explanation for our data. The model assumes that the observer accumulates noisy samples of evidence over time until a decision bound is reached, which determines their choice and reaction time. This process can be seen as a dynamic extension of the perceptual process described in Equation 1,

(6) $$\begin{array}{c} s_{t+\Delta t}=s_{t}+\;\mu_{s}+\;\varepsilon_{s,t+\Delta t},\; \end{array}$$

where *s_t_* is the accumulated evidence up to time *t*, which is described as evolving over small time steps, Δ*t*, with added signal, µ_*s*_, and noise, ε_*s*,*t* + Δ*t*_, drawn from independent identically distributed Gaussian distributions with zero mean and variance $\sigma_{s}^{2}$. Example evidence accumulation traces are shown in Figure 5A (left), simulating the different evidence strengths (signal-to-noise ratios) used in these experiments. The observer commits to a decision when the accumulated evidence reaches a decision bound (deciding “right” when the evidence reaches the upper bound, or “left” at the lower bound; black curves on Figure 5A describe collapsing decision bounds, which account for how the observer commits to a decision based on little evidence without very long decision times). The response time includes additional nondecision time (e.g., the time from committing to a decision to planning and executing the button press response to report the decision).

Two-stage dynamic signal detection Theory suggests that the observer continues accumulating evidence after the bound, for a certain period of time, and this final accumulated evidence is used to judge decision confidence. This continued accumulation would contribute to confidence boost (additional evidence used for confidence that was not used for the perceptual decision, so long as there is greater signal than noise). With a short duration from stimulus offset to response cue, the maximum time an observer would have to continue accumulating evidence for confidence after their decision is the time to plan and execute the button press response (nondecision time) plus the inter-trial interval (200 ms; pink and purple bars in Figure 5B, top), at which point the observer would need to prepare to accumulate evidence for the next decision. The median postdecision time in the short duration to response cue condition described by the distance from the green to the red line in Figure 5A (left). With a long duration from stimulus offset to response cue, there is additional postdecision time (yellow bar of Figure 5B, bottom; distance from the vertical green to black line in Figure 5A). In the long duration condition, the postdecision time depends on the decision time: participants who decide sooner have more time to wait before entering their response (comparing fast with median in Figure 5B bottom), whereas the “poststimulus time” is the same. The long duration condition therefore distinguishes the postdecision time from the “poststimulus time.”

Classic two-stage dynamic signal detection theory suggests that the postdecision evidence accumulation continues with the same signal-to-noise ratio as prior to decision commitment (Figure 5A top left). This would predict that with longer postdecision time the distributions of accumulated evidence from different underlying stimulus strengths become more separable, leading to better confidence efficiency with longer postdecision evidence accumulation (Figure 5A top and right filled bars). In these experiments, the stimulus was removed after 400 ms, meaning the observer would need to maintain a representation of the stimulus to continue accumulating evidence. This representation likely decays over time, or in the extreme, is not present at all, meaning the observer only accumulates noise in the postdecision time period (Fig 5A. bottom left). This would only increase the noise with more time, predicting worse confidence efficiency with longer durations of postdecision evidence accumulation (Figure 5A right, open bars).

The predictions of Figure 5A suggest that confidence efficiency can improve with longer durations of postdecision time with some maintenance of the signal in ongoing accumulation (top row of Figure 5A left, filled bars to the right; as in the high-level task, Experiment 4; Figs. 4A, 4B), whereas if the ongoing signal decayed to be overwhelmed by noise, confidence efficiency would worsen with more time (bottom row of Figure 5A left, open bars to the right; as in the low-level task, Experiment 4; Figs. 4A, 4B). This model may, therefore, offer some theoretical basis for the postdecision changes in confidence efficiency we observe in these experiments, if the signal-to-noise ratio of the high-level perceptual evidence is relatively well maintained for ongoing accumulation (filled bars of Figure 5A, right) but rapidly decays for low-level perceptual evidence (open bars of Figure 5A, right).

A stricter prediction is that the signal-to-noise ratio of postdecision evidence accumulation depends on the predecision signal-to-noise ratio with short durations of postdecision accumulation. To test this finding, we ran an exploratory (not preregistered) analysis where we split participants in Experiment 2 into three equal groups (RT groups; 30 participants each) based on their median reaction time. We found that perceptual sensitivity did not substantially differ across RT groups nor between tasks within RT groups (minimum BF_01_ = 1.98), suggesting that those participants who took longer to respond were accumulating evidence with a lower signal-to-noise ratio for a longer duration. In other words, participants who took longer to respond had a lower predecision signal-to-noise ratio and so should experience relatively less benefit from ongoing accumulation in the high-level task, and relatively less harm from ongoing accumulation in the low-level task. The number of trials in each group was only sufficient to estimate confidence efficiency, not to fit the full model with confidence noise and boost (Mamassian & de Gardelle, 2022). The difference between confidence efficiency in the high-level and low-level tasks was dependent on the median reaction time (Figures 5C, 5D): for the fast responders, confidence efficiency was greater in the high-level task (*E*(δ | *x*_*high* − *low*_) = 0.71 [0.33; 1.12]; BF_10_ > 1,000); for the median responders, confidence efficiency was slightly greater in the low-level task (*E*(δ | *x*_*high* − *low*_) = −0.44 [−0.81; −0.07]; BF_10_ = 3.03); and for the slow responders, confidence efficiency was even greater in the low-level task (*E*(δ | *x*_*high* − *low*_) = −0.89 [−1.30; −0.49]; BF_10_ > 1,000). This pattern of effects was predicted by simulating a model (Figure 5E) in which confidence efficiency in the high-level task was predicted by continued accumulation with the same signal-to-noise ratio as prior to decision commitment, whereas in the low-level task observers only accumulated additional noise (the red bars of Figure 5A correspond with the medium response time observers, the middle dot of Figure 5E). Across RT groups, confidence efficiency decreased in the high-level task with increasing reaction time, whereas in the low-level task confidence efficiency slightly increased with increasing reaction time.

Another prediction is that the difference in confidence efficiency between the short and long durations to response cue conditions would depend on decision time, such that participants with faster reaction times in the short condition (indicating faster decision times) would have a comparatively longer postdecision time in the long condition compared with those who take longer to decide (Figure 5B). As an exploratory analysis, we split participants in Experiment 4 based on their reaction times in the short duration condition (Figure 5F, left column). In the low-level task observers with faster response times showed better confidence efficiency in the short compared with the long condition (*E*(δ | *x*_*long* − *short*_) = 0.81 [0.36; 1.23]; BF_10_ > 1,000), whereas observers with median and long response times showed the opposite effect (median responders: *E*(δ | *x*_*long* − *short*_) = −1.03 [−1.49; −0.59]; BF_10_ >1 0,00) slow responders: *E*(δ | *x*_*long* − *short*_) = −0.92 [−1.35; −0.48]; BF_10_ > 1,000) (summary in Figure 5G). The opposite pattern was visible in the high-level task: fast responders showed worse confidence efficiency in the short condition (*E*(δ | *x*_*long* − *short*_) = −1.54 [−2.01; −1]; BF_10_ > 1,000), whereas median and slow responders showed better confidence efficiency in the short condition (median responders: *E*(δ | *x*_*long* − *short*_) = 2.20 [1.55; 2.88]; BF_10_ > 1,000; slow responders: *E*(δ | *x*_*long* − *short*_) = 0.56 [0.167; 0.94]; BF_10_ = 6.54) (Figure 5G). Note that a simple effect of “poststimulus time” would predict no difference in confidence efficiency in the long condition dependent on decision time in the short condition (poststimulus time is the same for the three groups of participants). Instead, we find confidence efficiency is modulated across decision-time groups in the long condition, suggesting the effect is driven by underlying “postdecision time.”

In Experiment 5, we tested whether these patterns of behavior generalize to other stimuli, as an indicator that the level of visual processing is driving the interaction (as opposed to some property particular to the avatar face stimuli or the perceptual tasks). In a high-level task observers judged whether a biological motion stimulus appeared to be walking to the left or right of directly toward them. In a low-level task, they judged whether two central dots of that stimulus were rotated clockwise or counterclockwise of vertical.

The results followed a similar pattern as those found in Experiment 4, although weaker (Figure 5G). For brevity, we present only the analysis of participants split by response times. In the low-level task, the pattern of effects were similar across experiments (Figure 5G, top), but in Experiment 5 fast and median responders showed no substantial difference in confidence efficiency (fast responders: *E*(δ | *x*_*long* − *short*_) = 0.31 [−0.06; 0.69]; BF_10_ = 0.66); median responders: *E*(δ | *x*_*long* − *short*_) = −0.27 [−0.66; 0.1]; BF_10_ = 0.50; slow responders: (*E*(δ | *x*_*long* − *short*_) = −0.422 [−0.79; −0.05]; BF_10_ = 2.17). In the high-level task, the switch from short condition showing better confidence efficiency to long condition showing better confidence efficiency was delayed to the median and slow responders (Figure 5G, bottom; confidence efficiency was the same across conditions for fast responders: *E*(δ | *x*_*long* − *short*_) = −0.01 [−0.38; 0.37]; BF_10_ = 0.21; median responders: (*E*(δ | *x*_*long* − *short*_) = −0.44 [−0.80; −0.05]; BF_10_ = 2.00; and slow responders: (*E*(δ | *x*_*long* − *short*_) = 0.85 [0.42; 1.27]; BF_10_ > 1,000).

## Discussion

We found differences in confidence efficiency within observers making different perceptual decisions about the same visual stimuli (despite similar perceptual sensitivity and reaction times across perceptual decisions). In Experiment 1, we found greater confidence efficiency for high-level compared with low-level decisions, as predicted by reverse hierarchy theory (Hochstein & Ahissar, 2002). These findings were reversed in Experiment 2, when the white noise mask was removed and there was a short duration from stimulus offset to response cue. We explained this reversal in Experiments 3 and 4, showing both an effect of backward masking on low-level confidence efficiency, and an effect of postdecision time. We generalized these results to different stimuli in Experiment 5, showing the results are not driven by something particular to the stimuli, but relate to the hierarchical level of visual representation. This highlights how ongoing processes for confidence (Yu, Pleskac, & Zeigenfuse, 2015; Desender, Vermeylen, & Verguts; 2022) can be extended for relatively long durations, even though these ongoing processes may be detrimental to the quality of confidence decisions. Especially for low-level representations, confidence would benefit from terminating ongoing processes before evidence degradation. This brings into question what mechanisms are responsible for finalizing confidence: optimal processing would require some control of how much additional accumulation contributes to confidence decisions (perhaps through nested cognitive processes) (Recht, Jovanovic, Mamassian, & Balsdon, 2022).

Although we did not find the simple main effect that was predicted from reverse hierarchy theory (Hochstein & Ahissar, 2002), these results are not inconsistent with its general principles: confidence in the low-level task was much more vulnerable to noise than confidence in the high-level task. Our findings further suggest that high-level perceptual representations can persist for relatively longer durations. If this is also a quality of hierarchical organization, then high-level representations should also be less permeable to degradation in other tasks requiring long maintenance times, such as visual working memory tasks (as has recently been suggested) (Ding, Cueva, Tsodyks, & Qian, 2017; Luu, Zhang, Tsodyks, & Qian, 2022). In this way, high-level representations would not only be more readily accessible for explicit scrutiny, but also more readily available over time.

We suggest that the interaction between reaction time and level of visual processing on confidence efficiency can be explained by the dynamics of ongoing evidence accumulation processes, such as those suggested by the two-stage dynamic signal detection theory framework (Pleskac & Busemeyer, 2010). The difference in the effect of postdecision time on high-level and low-level representations can be captured by assuming the ongoing perceptual representation degrades over time and, for low-level representations, quickly becomes overwhelmed by additional noise. These dynamic changes in the signal-to-noise ratio of the continued accumulation for confidence may appear in contradiction with Yu et al. (2015), who found a constant accumulation rate. However, this difference could be explained by differences in experimental design, for example, signal degradation could have been reduced in this earlier work by continuously presenting the stimulus until the confidence decision. Further investigation is required to characterize the nature of these dynamic changes in the signal-to-noise ratio. Our results suggest there is both signal degradation as well as additional noise. Some of this noise may accumulate over time in a manner proportional to perceptual noise, this would explain how fast responders (who have less perceptual noise) show better confidence efficiency after a longer duration in the low-level task. This finding is also consistent with the measured confidence boost, which tended to increase with duration in the high-level task, but rarely rose above 0 in the low-level task (unless continued processing was cut-off early, limiting accumulation noise). However, simulated confidence efficiency with only additional perceptual noise was still superior to that measured from human behavior (Figure 5A), suggesting a second source of noise in later processing stages, such as converting the confidence representation into the appropriate behavioral response.

These dynamic changes in the quality of accumulated evidence are linked with the level of representation in the visual hierarchy, as evidenced by the similar pattern of results in Experiments 4 and 5 (which used different stimuli). High-level representations must be maintained for a long duration (as all but the fast responders showed better confidence efficiency after longer durations) and this duration must be relative to the time of decision commitment (because the poststimulus time is approximately the same across groups in the long-duration condition). Although for high-level representations this conservation over time could be linked with a more conceptual representation, the effect of visual backward masking on low-level confidence efficiency suggests a strong influence of ongoing perceptual processes to metacognition. This effect of the mask could be described as injecting even more noise into the ongoing low-level accumulation. Further investigation is required to more finely map these dynamics across the visual hierarchy and understand the sources of additional noise at the neural level. Overall, these results highlight how ongoing perceptual processes continue to contribute to metacognition long after perceptual decision commitment.

Although we suggest that the two-stage dynamic signal detection theory framework provides a useful explanation of the pattern of results in these experiments, more work is required to test the theoretical predictions explicitly in light of this new evidence. Moreover, in our basic formulation, we make several simplifying assumptions, such as the assumption that observers might continue to accumulate evidence for confidence until the start of the next decision. Although the effect of the mask suggests additional accumulation for confidence continues for more than 400 ms after stimulus offset, the current design cannot be used to test when it actually ends. Another factor is how continued accumulation might proceed in other tasks, or in designs where participants are asked for a confidence rating after every perceptual decision.

In summary, our ability to evaluate the accuracy of our perceptual decisions using confidence is strongly reliant on ongoing perceptual representations. Our results suggest that this continued perceptual processing can be extended for relatively long durations, even though the quality of the perceptual representation declines over time. The dynamics of ongoing perceptual representations can explain differences in confidence efficiency despite no differences in perceptual sensitivity, suggesting that perceptual sensitivity alone is not sufficient to explain all perceptual effects on metacognitive processes.

## Footnotes

1 Specific pages for each Experiment pre-registration: Experiment 1: https://osf.io/fs5wr/ Experiment 2: https://osf.io/qwbke/ Experiment 3: low-level: https://osf.io/s9dtu/ high-level: https://osf.io/tw8zy/ Experiment 4: https://osf.io/gn9f5/ Experiment 5: https://osf.io/czxep/
